# The influence of glycemic status on the performance of cystatin C for acute kidney injury detection in the critically ill

**DOI:** 10.1080/0886022X.2019.1586722

**Published:** 2019-04-03

**Authors:** Yujun Deng, Lin Wang, Yating Hou, Jianchao Ma, Ruibin Chi, Heng Ye, Yiling Zhai, Danqing Zhang, Lu Gao, Linhui Hu, Tieying Hou, Jinghua Li, Ning Tan, Chunbo Chen

**Affiliations:** a The Second School of Clinical Medicine, Southern Medical University, Guangzhou, People's Republic of China;; b Department of Critical Care Medicine, Guangdong Provincial People's Hospital, Guangdong Academy of Medical Sciences, Guangzhou, People’s Republic of China;; c Shantou University Medical College, Shantou, People's Republic of China;; d Department of Nephrology, Guangdong Provincial People's Hospital, Guangdong Academy of Medical Sciences, Guangzhou, People’s Republic of China;; e Department of Critical Care Medicine, Xiaolan Hospital of Southern Medical University, Zhongshan, People’s Republic of China;; f Department of Critical Care Medicine, Guangzhou Nansha Central Hospital, Nansha, People’s Republic of China;; g Department of Clinical Laboratory, Guangdong Provincial People's Hospital, Guangdong Academy of Medical Sciences, Guangzhou, People’s Republic of China;; h Department of Cardiology, Guangdong Cardiovascular Institute, Guangdong Key Laboratory of Coronary Disease, Guangdong Provincial People's Hospital, Guangdong Academy of Medical Sciences, Guangzhou, People's Republic of China;; i National Clinical Research Center for Kidney Disease, State Key Laboratory of Organ Failure Research, Nanfang Hospital, Southern Medical University, Guangzhou, P.R. China

**Keywords:** Serum cystatin C, acute kidney injury, glycated hemoglobin, diabetes mellitus, prediabetes

## Abstract

**Objective:** Serum cystatin C (sCysC) used clinically for detecting early acute kidney injury (AKI) was reported to be independently associated with hemoglobin (HbA1c) levels, diabetes, and prediabetes. We aimed to assess the influence of HbA1c levels, diabetes, or prediabetes on the performance of sCysC for AKI detection in critically ill adults.

**Methods:** A prospective observational study was conducted in a mixed medical-surgical intensive care unit (ICU). Patients were divided into four quartiles based on levels of HbA1c or serum glucose at ICU admission, respectively. Additionally, patients were stratified into four subgroups according to HbA1c levels and history of diabetes, namely recognized diabetes (previous diagnosis of diabetes), unrecognized diabetes, prediabetes, and normal glycemic status. Comparisons were made using the area under the receiver operator characteristic curve (AUC) for AKI detection, and reassessed after patient stratification by above-mentioned glycemic status.

**Results:** Multivariable linear regression revealed that HbA1c levels and history of diabetes were positively related with sCysC (all *p* < .05). Although stratification for above-mentioned glycemic status displayed no significant difference between AUC of sCysC (all *p* > .05), sCysC yielded the highest AUCs for detecting AKI in diabetic patients. Moreover, higher optimal cutoff values of sCysC to detect AKI were observed in patients with versus without diabetes.

**Conclusion:** Glycemic status has no significant impact on the accuracy of sCysC for AKI detection in critically ill adults and a higher optimal cutoff value of sCysC for AKI detection should be considered in diabetic patients.

## Introduction

Acute kidney injury (AKI) is prevalent and independently associated with adverse outcomes [1,2], especially in the critically ill [1,3]. The unavoidable delay in the identification of AKI has stimulated the development of several novel biomarkers, such as neutrophil gelatinase-associated lipocalin [4], kidney injury molecule-1 [5], tissue inhibitor of metalloproteinase-2, insulin-like growth factor binding protein 7 [3], and cystatin C (CysC) [6–8]. Among them, serum cystatin C (sCysC) is clinically available in European, North American, and Asian medical centers. Since clinically available markers can be applicable worldwide [9], sCysC becomes one of the most extensively studied AKI biomarkers [6,10,11]. CysC, a 13-kDa cysteine proteinase inhibitor [12], is freely filtered in the glomeruli, completely absorbed in the renal proximal tubule and is not secreted into the urine by the tubule [10,12]. The characteristics of CysC make it a reliable marker of the glomerular filtration rate (GFR) [12,13]. Although previous studies suggested that CysC may be a potential AKI biomarker [7,10,14], the inconsistent results [14–16] limit its widespread application for AKI detection in clinical practice.

Apart from AKI, elevated levels of sCysC are independently associated with diabetes mellitus (DM) [17,18], and prediabetes [19,20]. Notably, the previous study [17] revealed that DM caused blood CysC to deviate higher than expected from GFR. In addition, glycated hemoglobin (HbA1c) was found to be an independent predictor for the increment of sCyC levels in diabetic patients [21]. Moreover, diabetes, prediabetes and elevated levels of HbA1c are common in critically ill adults [22–25]. Importantly, critically ill patients with diabetes or high levels of HbA1c are at a high risk of developing AKI [25,26]. However, the impact of HbA1c levels, diabetes, or prediabetes on the performance of sCysC for AKI detection in heterogeneous patient populations has not been well clarified.

Accordingly, we undertook a prospective, observational study in a large population of a mixed medical–surgical intensive care unit (ICU) to evaluate the performance characteristic of sCysC for AKI detection in patients stratified for glycemic status. HbA1c levels, serum glucose levels at ICU admission, or history of diabetes were used as indicators of glycemic status.

## Methods

### Study design and participants

The present prospective observational study was conducted in mixed medical–surgical ICU of a tertiary care hospital in China. All consecutive patients aged 18 years or older between October 2014 and June 2016 were eligible for enrollment. The exclusion criteria included end-stage renal disease (ESRD) or undergoing renal replacement therapy (RRT) before ICU admission, history of nephrectomy or kidney transplantation before ICU admission, or refusal to consent. The protocol met the Strengthening the Reporting of Observational Studies in Epidemiology [27] and Standards for Reporting Diagnostic Accuracy [28] criteria. All experiments were performed in accordance with the approved guidelines, protocols, and regulations. The Ethics Committee of the Guangdong Provincial People's Hospital approved the protocol. Written informed consent was obtained from each patient or from appropriate surrogates for patients unable to consent.

### Data collection

Patients’ baseline clinical characteristics were prospectively collected. All samples were collected simultaneously within one hour after patients were admitted to the ICU. Serum CysC, and HbA1c levels were measured once at ICU admission. Serum creatinine (sCr) and glucose were measured at ICU admission, and thereafter at least once a day as a part of routine clinical care during ICU hospitalization. Patient’s hourly urine output from ICU admission to discharge was recorded. The following clinical variables were collected: age, sex, body mass index (BMI), hypertension, DM, chronic kidney disease (CKD), coronary artery disease (CAD), malignance, thyroid disease, sepsis, previous application of antidiabetic drugs, previous corticosteroids administration, admission type, baseline sCr, baseline-estimated glomerular filtration rate (eGFR), sCr at ICU admission, Acute Physiology and Chronic Health Evaluation (APACHE II) score, length of ICU stay, length of hospital stay, renal RRT during ICU stay, ICU mortality, and in-hospital mortality. The baseline eGFR was estimated by the Modification of Diet in Renal Disease (MDRD) formula [29].

### Definitions

AKI was diagnosed based on the Kidney Disease Improving Global Outcomes (KDIGO) criteria for AKI within 1 week after ICU admission [30] as any of the following: increase in sCr by ≥0.3 mg/dl (≥26.5 µmol/l) within 48 h, increase in sCr to ≥1.5 times baseline within 1 week, or urine output <0.5 mL/kg/h for 6 h. A baseline creatinine was determined according to the following rules ranked in the descending order of preference as previously described [31]: (1) the most recent pre-ICU value between 30 and 365 days before ICU admission (*n* = 148); (2) a stable pre-ICU value >365 days for patients aged <40 years (stable defined as within 15% of the lowest ICU measurement) before ICU admission (*n* = 4); (3) pre-ICU value >365 days before ICU admission and less than the initial sCr at ICU admission (*n* = 56); (4) a pre-ICU value (between 3 and 39 days before ICU admission) less than or equal to the initial on-admission sCr to ICU and not distinctly in AKI (*n* = 647); (5) the lowest sCr upon initial admission to ICU (*n* = 150), the last ICU value (*n* = 183), or the minimum value at follow-up to 365 days (*n* = 129). Established AKI was defined as diagnosis of AKI at ICU admission. Later-onset AKI indicated as no AKI diagnosis at ICU admission but reached the KDIGO criteria within 1 week after admission. Sepsis was diagnosed based on the American College of Chest Physicians and the Society of Critical Care Medicine Consensus Conference Committee guidelines [32].

### Measurement of serum CysC, creatinine and HbA1c

Serum CysC, creatinine, and glucose were measured using the UniCel DxC 800 Synchron System (Beckman Coulter, Brea, CA, USA) according to the manufacturer’s instructions. The coefficients of inter-assay and intra-assay variation in sCysC were <5% and ≤10%, respectively. The stability of sCysC has already been demonstrated [33,34], and thereby pre-analysis about the influence of cooling or freezing on the samples was not executed. Measurement of HbA1c was performed using the ion-exchange high-performance liquid chromatography (HPLC) on the Bio-Rad D-10 system (Bio-Rad Laboratories, Hercules, CA) according to the manufacturer’s instructions. The normal range was 4.3% to 6.1%. All samples were measured at the central laboratory of the Guangdong Provincial People's Hospital using a standard protocol within 24 h after collection. The personnel measuring were blinded to each patient’s clinical characteristics.

### Patient groups

We first categorized patients into four quartiles based on admission HbA1c levels. The patients were also divided into four quartiles according to admission serum glucose levels. Additionally, stratification analysis was performed based on HbA1c levels and history of diabetes to elucidate potential misclassification of a formerly undiagnosed abnormal glycemic status (diabetes or prediabetes). Thereby, the patients were stratified into four subgroups analogous to the study by Kompoti et al. [24], namely normal glycemic status, prediabetes, recognized diabetes, and unrecognized diabetes. According to the patients’ prior hospital case records and histories provided by the patients or their family, patients were considered as having ‘recognized diabetes’ if they had a diagnosis of diabetes before ICU admission or at least 1 prescription for an oral antidiabetic agent or insulin before ICU admission. Based on the American Diabetes Association criteria [35], patients without previous diagnosis of DM were further classified into patients with ‘unrecognized diabetes’ (with HbA1c ≥ 6.5%), patients with ‘prediabetes’ (HbA1c within the range 5.7% to 6.4%), and patients with ‘normal glycemic status’ (with HbA1c < 5.7%). Baseline characteristics were compared among these groups.

### Statistical analysis

We used the SPSS version 13.0 (SPSS, Chicago, IL), MedCalc version 12.5.0 (MedCalc Software, Ostend, Belgium), and R version 3.3.1 (R Foundation for Statistical Computing, Vienna, Austria) software programs for statistical analysis.

Based on a chart review of 177 patients admitted to ICU (unpublished), the occurrences of total AKI and later-onset AKI was 19.2% and 8.9%, respectively. According to the previous study described by Hanley et al. [36], we estimated that a sample size of 1040 patients was required, with a two-sided test (α error = 5%; power = 80%). Continuous variables were presented as means ± standard deviation or medians (interquartile range). Categorical variables were expressed as number (percentage). The non-normally distributed continuous variables were compared using Wilcoxon rank-sum test or the Kruskal–Wallis test for one-way analysis of variance. If the Kruskal–Wallis test showed statistical significance, a *post hoc* Steel–Dwass test was subsequently conducted. To compare the categorical variables, the Chi-square or Fisher’s exact test was used. The variables (sCysC, serum glucose, sCr, HbA1c, and baseline eGFR) displayed non-normal distributions. Therefore, a nonparametric test (Spearman’s) was used to assess their correlations. Multivariable linear regression analysis with a stepwise variable selection was then used to assess the relationship between sCysC and other variables.

In order to assess the discrimination capability of sCysC for AKI detection, receiver-operating characteristic (ROC) curve was generated. The area under the curve (AUC) was derived from the ROC curve. All confidence interval (CI) presented are 95%. The comparison of AUC between the groups was conducted using Hanley–McNeil methods [37]. AUCs were described with the following values as previously described [38]: 0.90–1.0 excellent, 0.80–0.89 good, 0.70–0.79 fair, 0.60–0.69 poor and 0.50–0.59 no useful performance. The sensitivity and specificity of sCysC was calculated. Based on the Youden’s index [39], a statistically derived value, maximizing the sum of the sensitivity and specificity was used to define the optimal cutoff value for AKI detection. All the tests were two-tailed, and *p* < .05 was considered statistically significant.

## Results

### Patient characteristics

**Figure 1.**
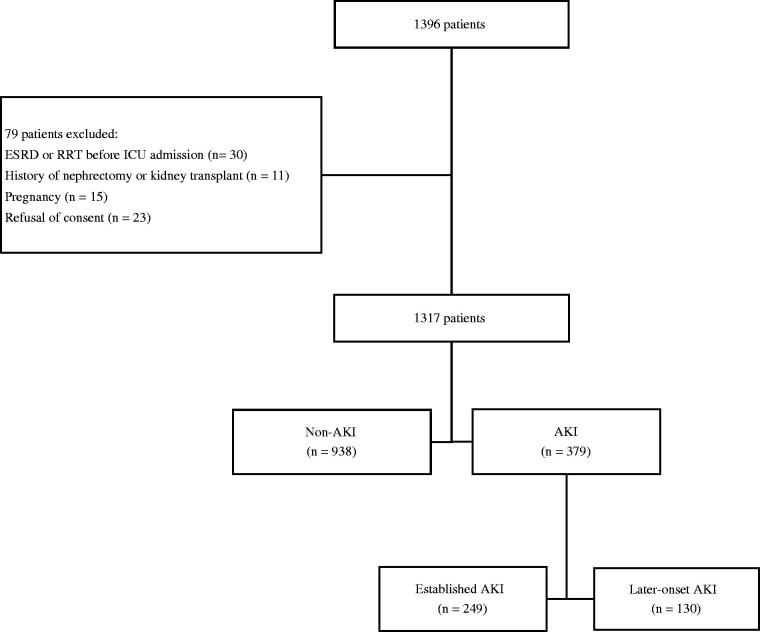
Recruitment of patients into the study. AKI: acute kidney injury; ESRD: end-stage renal disease; ICU: intensive care unit; RRT: renal replacement therapy. Established AKI indicated the diagnosis of AKI at ICU admission. Later-onset AKI was defined as no AKI diagnosis at ICU admission but reaching the KDIGO criteria within 1 week after admission.

Of 1317 enrolled patients, 379 (28.8%) patients were diagnosed as AKI (Figure 1). There were 225 diabetic patients including patients with a prior diagnosis of DM and those with admission HbA1c ≥ 6.5% but no prior diagnosis of DM (Additional file 1: Supplementary Table S1). A greater percentage of the patients with AKI had preexisting DM and CKD compared to patients without AKI (all *p* < .05). The concentrations of sCysC, sCr, serum glucose, and HbA1c at ICU admission were significantly higher in patients with AKI than in non-AKI patients. Higher BMI and APACHE II scores were also recorded in patients with versus without AKI. Moreover, adverse outcomes occurred more frequently in patients with AKI than in those without AKI.

The results revealed weak but significant correlations between sCysC and HbA1c (*r* = 0.224, *p* < .001) and between sCysC and history of diabetes (*r* = 0.190, *p* < .001), but not between sCysC and admission serum glucose (*r* = 0.008, *p* = .759) (Table 1). In multivariable linear regression (Table 2), HbA1c at ICU admission (standardized beta = 0.042, *p* = .039) and history of diabetes (standardized beta = 0.070, *p* < .001) were positively related to sCysC.

**Table 1.** Factors associated with sCysC by bivariate correlation analysis.

Variables	sCysC at ICU admission, mg/L	
	Spearman’s rho	*p*
Age, years	0.398	<.001
Males, n (%)	0.237	<.001
BMI, kg/m^2^	0.053	.056
History of diabetes, n (%)	0.190	<.001
CKD, n (%)	0.373	<.001
Malignancies, n (%)	0.127	<.001
Previous use of corticosteroids, n (%)	0.125	<.001
APACHE II	0.390	<.001
HbA1c at ICU admission, %	0.224	<.001
Serum glucose at ICU admission, mg/dL	0.008	.759
sCr at ICU admission, mg/dL	0.530	<.001

APACHE II: Acute Physiology and Chronic Health Evaluation score; BMI: body mass index; CKD: chronic kidney disease, defined as baseline eGFR <60 mL/min/1.73 m^2^; eGFR: estimated glomerular filtration rate; HbA1c: glycosylated hemoglobin; ICU: intensive care unit; sCr: serum creatinine; sCysC: serum cystatin C.

**Table 2.** Multivariable linear regression analysis with sCysC as dependent variable.

	sCysC at ICU admission, mg/L	
Independent variables^a^	Standardized ß	*p*
Age, years	0.140	<.001
Males, n (%)	−0.063	<.001
History of diabetes, n (%)	0.070	<.001
CKD, n (%)	0.098	<.001
Previous use of corticosteroids, n (%)	0.083	<.001
APACHE II	0.157	<.001
sCr at ICU admission, mg/dL	0.650	<.001
Serum glucose at ICU admission, mg/dL	−0.140	<.001
HbA1c at ICU admission, %	0.042	.039
Constant	0.031 (Unstandardized)	.600

APACHE II: Acute Physiology and Chronic Health Evaluation score; BMI: body mass index; CKD: chronic kidney disease, defined as baseline eGFR <60 mL/min/1.73 m^2^; eGFR: estimated glomerular filtration rate; HbA1c: glycosylated hemoglobin ICU: intensive care unit; sCr, serum creatinine; sCysC: serum cystatin C.

a Independent variables included age, males(sex), BMI, history of diabetes, CKD (baseline eGFR <60 mL/min/1.73 m^2^), malignancies, previous use of corticosteroids, APACHE II, sCr at ICU admission, serum glucose at ICU admission, HbA1c at ICU admission. Variables not listed in the table were removed from the stepwise analysis. Adjusted R square 0.673.

### Performance of sCysC for AKI detection

Serum CysC at ICU admission showed fair discriminative ability for total AKI and poor predictive ability for later-onset AKI overall (Additional file 2: Supplementary Table S2). The AUC of sCysC for detecting total AKI was 0.765 (95% CI, 0.736–0.795). In addition, sCysC predicted later-onset AKI with an AUC of 0.689 (95% CI, 0.641–0.738).

### Performance of sCysC for AKI detection in patients with different levels of HbA1c

Participants were initially divided into four quartiles based on HbA1c levels at ICU admission. The baseline clinical and laboratory characteristics with different concentrations of HbA1c are presented in Table 3. Serum CysC increased across HbA1c quartiles (*p* < .001). Patients in the highest quartile were older and had greater incidence of AKI compared to those in other quartiles.

**Table 3.** Characteristics of enrolled patients according to quartiles of HbA1c.

Variables	Quartile I	Quartile II	Quartile III	Quartile IV	*p*
**Entire cohort**					
Number	329	283	335	370	/
Age, years	45 (32–57)**^a^**	51 (39–63)**^b^**	56 (45–65)**^c^**	63 (54–71)**^d^**	<.001
Males, n (%)	176 (53.5)	155 (54.8)	180 (53.7)	209 (56.5)	.850
BMI, kg/m^2^	22.19 (20.83–23.07)**^e^**	22.19 (20.76–23.07)**^e^**	22.43 (21.22–24.03)	22.58 (21.72–25.07)	<.001
Total AKI, n (%)	68 (20.7)	68 (24.0)	86 (25.7)	157 (42.4)	<.001
History of diabetes, n (%)	2 (0.6)	2 (0.7)	12 (3.6)	99 (26.8)	<.001
CKD, n (%)	11 (3.3)	17 (6.0)	16 (4.8)	43 (11.6)	<.001
APACHE II	9 (7–14)**^e^**	10 (7–14)**^f^**	11 (8–17)**^f^**	13 (10–21)	<.001
sCr at ICU admission, mg/dL	0.80 (0.63–0.98)**^f^**	0.79 (0.68–1.00)**^f^**	0.83 (0.68–1.05)**^f^**	0.92 (0.71–1.18)	<.001
sCysC at ICU admission, mg/L	0.76 (0.61–0.96)**^e^**	0.81 (0.64–1.06)**^f^**	0.84 (0.67–1.07)**^f^**	0.95 (0.75–1.32)	<.001
Serum glucose at ICU admission, mg/dL	118.3 (101.5–138.4)**^e^**	120.4 (104.4–145.8)**^f^**	124.9 (106.2–151.0)**^f^**	148.0 (119.3–191.2)	<.001
HbA1c at ICU admission, %	5.1 (4.9–5.3)**^a^**	5.5 (5.4–5.6)**^b^**	5.8 (5.7–5.9)**^c^**	6.5 (6.2–7.1)**^d^**	<.001
**Patients without established AKI**					
Number	216	315	207	330	/
Age, years	44 (30–55)**^a^**	49 (38–61)**^b^**	56 (44–63)**^c^**	61 (52–69)**^d^**	<.001
Males, n (%)	106 (49.1)	169 (53.7)	104 (50.2)	175 (53.0)	.688
BMI, kg/m^2^	22.06 (20.41–22.99)**^f^**	22.19 (20.82–23.11)**^f^**	22.19 (20.80–23.66)**^f^**	22.59 (21.70–25.56)	<.001
Later-onset AKI, n (%)	22 (10.2)	33 (10.5)	13 (6.3)	62 (18.8)	<.001
History of diabetes, n (%)	2 (0.9)	0 (0.0)	4 (1.9)	61 (18.5)	<.001
CKD, n (%)	6 (2.8)	8 (2.5)	5 (2.4)	19 (5.8)	.079
APACHE II	9 (7–13)**^e^**	9 (7–13)**^e^**	10 (8–14)**^f^**	12 (9–16)	<.001
sCr at ICU admission, mg/dL	0.77 (0.62–0.93)	0.77 (0.66–0.94)	0.79 (0.67–0.94)	0.83 (0.67–0.98)	.078
sCysC at ICU admission, mg/L	0.73 (0.61–0.91)**^e^**	0.75 (0.61–0.96)**^f^**	0.81 (0.65–0.96)	0.86 (0.68–1.09)	<.001
Serum glucose at ICU admission, mg/dL	116.6 (100.7–135.9)**^f^**	119.3 (103.1–139.5)**^f^**	118.8 (104.0–147.1)**^f^**	138.7 (114.5–167.3)	<.001
HbA1c at ICU admission, %	5.0 (4.9-5.2)**^a^**	5.5 (5.4-5.6)**^b^**	5.8 (5.7–5.8)**^c^**	6.3 (6.1–6.7)**^d^**	<.001

APACHE II: Acute Physiology and Chronic Health Evaluation score; AKI: acute kidney injury; BMI: body mass index; CKD: chronic kidney disease, defined as baseline eGFR <60 mL/min/1.73 m^2^; eGFR: estimated glomerular filtration rate; HbA1c: glycosylated hemoglobin; ICU: intensive care unit; sCr: serum creatinine; sCysC: serum cystatin C; Established AKI, defined as diagnosis of AKI at ICU admission; Later-onset AKI, indicated no AKI diagnosis at ICU admission but reaching the KDIGO criteria within 1 week after admission.

The non-normally distributed continuous variables are expressed as median (25th percentile to 75th percentile [interquartile range]). Categorical variables are expressed as n (%).

Patients were stratified into 4 quartiles according to the HbA1c levels at ICU admission.

Total AKI: Quartile cut points for HbA1c were 5.4%, 5.7%, and 6.1%.

Later-onset AKI: Quartile cut points for HbA1c were 5.3%, 5.7%, and 6.0%.

^a^*p*<.05 vs. Quartile II, Quartile III, and Quartile IV; ^b^*p*<.05 vs. Quartile I, Quartile III, and Quartile IV; ^c^*p*<.05 vs. Quartile I, Quartile II, and Quartile IV; ^d^*p*<.05 vs. Quartile I, Quartile II, and Quartile III; ^e^*p*<.05 vs. Quartile III and Quartile IV; and ^f^*p*<.05 vs. Quartile IV.

The performance of sCysC in detecting AKI after stratification for HbA1c levels were assessed (Table 4). The AUC for total AKI detection was calculated as 0.762 in quartile I, 0.722 in quartile II, 0.727 in quartile III, and 0.788 in quartile IV. No significant difference in the AUC between any two subgroups was observed (all *p* > .05). Similarly, sCysC failed to yield any significantly different AUC for later-onset AKI prediction after patient stratification by HbA1c levels. The AUC for later-onset AKI prediction was calculated as 0.698 in quartile I, 0.655 in quartile II, 0.712 in quartile III, and 0.688 in quartile IV.

**Table 4.** Performance of sCysC in detecting AKI according to quartiles of HbA1c.

Group	AUC-ROC	95% CI	*P*	Cutoff (mg/L)	SENS	SPEC
Total AKI						
Quartile I	0.762 ± 0.034	0.696–0.828	<.001	0.83	0.721	0.690
Quartile II	0.722 ± 0.037	0.649–0.795	<.001	1.15	0.456	0.907
Quartile III	0.727 ± 0.033	0.662–0.793	<.001	0.90	0.686	0.679
Quartile IV	0.788 ± 0.024	0.740–0.835	<.001	1.03	0.688	0.775
Later-onset AKI						
Quartile I	0.698 ± 0.059	0.582–0.813	.002	0.84	0.682	0.686
Quartile II	0.655 ± 0.048	0.561–0.748	.004	0.73	0.758	0.475
Quartile III	0.712 ± 0.045	0.606–0.819	.010	0.88	0.769	0.624
Quartile IV	0.688 ± 0.040	0.610–0.766	<.001	1.04	0.532	0.784

AKI: acute kidney injury; AUC-ROC: area under the receiver operating characteristic curve; CI: confidence interval; HbA1c: glycosylated hemoglobin; sCysC: serum cystatin C; SENS: sensitivity; SPEC: specificity.

Patients were stratified into 4 quartiles according to the HbA1c levels at ICU admission.

Total AKI: quartile cut points for HbA1c were 5.4%, 5.7%, and 6.1%.

AUC of Quartile I vs. AUC of Quartile II, Z = 0.796, *p* = .426.

AUC of Quartile I vs. AUC of Quartile III, Z = 0.739, *p* = .460.

AUC of Quartile I vs. AUC of Quartile IV, Z = 0.625, *p* = .532.

AUC of Quartile II vs. AUC of Quartile III, Z = 0.101, *p* = .920.

AUC of Quartile II vs. AUC of Quartile IV, Z = 1.497, *p* = .135.

AUC of Quartile III vs. AUC of Quartile IV, Z = 1.495, *p* = .135.

Later-onset AKI: quartile cut points for HbA1c were 5.3%, 5.7%, and 6.0%.

AUC of Quartile I vs. AUC of Quartile II, Z = 0.565, *p* = .572.

AUC of Quartile I vs. AUC of Quartile III, Z = 0.189, *p* = .850.

AUC of Quartile I vs. AUC of Quartile IV, Z = 0.140, *p* = .888.

AUC of Quartile II vs. AUC of Quartile III, Z = 0.866, *p* = .386.

AUC of Quartile II vs. AUC of Quartile IV, Z = 0.528, *p* = .597.

AUC of Quartile III vs. AUC of Quartile IV, Z = 0.399, *p* = .690.

### Performance of sCysC for AKI detection in patients with different levels of serum glucose at ICU admission

After stratification by serum glucose at ICU admission, sCysC did not show significantly changed performance for AKI detection (Additional file 3: Supplementary Table S3). The levels of sCysC did not decrease across admission serum glucose quartiles (Additional file 4: Supplementary Table S4 and Additional file 5: Supplementary Table S5).

### Performance of sCysC for AKI detection after patient stratification by history of diabetes and HbA1c levels

Although Table 5 demonstrates no significant difference in the AUC of sCysC for AKI detection between any two subgroups (all *p* > .05), sCysC yielded the highest AUCs in diabetic patients (both recognized and unrecognized diabetes). In diabetic subgroup, sCysC showed good discrimination for total AKI and fair predictive ability for later-onset AKI. In patients with recognized diabetes, sCysC detected total AKI detection with an AUC of 0.816 (95% CI, 0.738–0.894) and predicted later-onset AKI with an AUC of 0.774 (95% CI, 0.632–0.917), respectively. In the unrecognized diabetes subgroup, sCysC detected total AKI detection with an AUC of 0.819 (95% CI, 0.735–0.903) and predicted later-onset AKI with an AUC of 0.750 (95% CI, 0.615–0.885), respectively.

**Table 5.** Performance of sCysC in detecting AKI according to HbA1c levels and history of diabetes.

Group	AUC-ROC	95% CI	*p*	Cutoff (mg/L)	SENS	SPEC
Total AKI						
I (Recognized diabetes)	0.816 ± 0.040	0.738–0.894	<.001	1.24	0.710	0.868
II (Unrecognized diabetes)	0.819 ± 0.043	0.735–0.903	<.001	1.31	0.545	0.985
III (Prediabetes)	0.728 ± 0.026	0.677–0.780	<.001	0.98	0.579	0.765
IV (Normal glycemic status)	0.738 ± 0.025	0.688–0.787	<.001	0.83	0.692	0.638
Later-onset AKI						
I (Recognized diabetes)	0.774 ± 0.073	0.632–0.917	.002	1.20	0.643	0.830
II (Unrecognized diabetes)	0.750 ± 0.069	0.615–0.885	.005	0.93	0.692	0.727
III (Prediabetes)	0.664 ± 0.043	0.580–0.748	< .001	0.87	0.653	0.596
IV (Normal glycemic status)	0.665 ± 0.037	0.592–0.738	< .001	0.72	0.796	0.465

AKI: acute kidney injury; AUC-ROC: area under the receiver operating characteristic curve; CI: confidence interval; HbA1c: glycosylated hemoglobin; SENS: Sensitivity; sCysC: serum cystatin C; SPEC: specificity; Later-onset AKI, indicated no AKI diagnosis at ICU admission but reaching the KDIGO criteria within 1 week after admission.

The ‘recognized diabetes’ was identified using the hospital case records and history provided by patients or their family; patients without previous diagnosis of diabetes were further classified according to the level of HbA1c at ICU admission as ‘unrecognized diabetes’ (HbA1c ≥6.5%), ‘prediabetes’ (HbA1c within the range 5.7% to 6.4%), and ‘normal glycemic status’ (HbA1c < 5.7%).

Total AKI: AUC of Group I vs. AUC of Group II, Z = 0.051, *p* = 0.959.

AUC of Group I vs. AUC of Group III, Z = 1.845, *p* = 0.065.

AUC of Group I vs. AUC of Group IV, Z = 1.654, *p* = 0.098.

AUC of Group II vs. AUC of Group III, Z = 1.811, *p* = 0.070.

AUC of Group II vs. AUC of Group IV, Z = 1.628, *p* = 0.103.

AUC of Group III vs. AUC of Group IV, Z = 0.277, *p* = 0.782.

Later-onset AKI: AUC of Group I vs. AUC of Group II, Z = 0.239, *p* = 0.811.

AUC of Group I vs. AUC of Group III, Z = 1.298, *p* = 0.194.

AUC of Group I vs. AUC of Group IV, Z = 1.332, *p* = 0.183.

AUC of Group II vs. AUC of Group III, Z = 1.058, *p* = 0.290.

AUC of Group II vs. AUC of Group IV, Z = 1.086, *p* = 0.278.

AUC of Group III vs. AUC of Group IV, Z = 0.018, *p* = 0.986.

**Figure 2.**
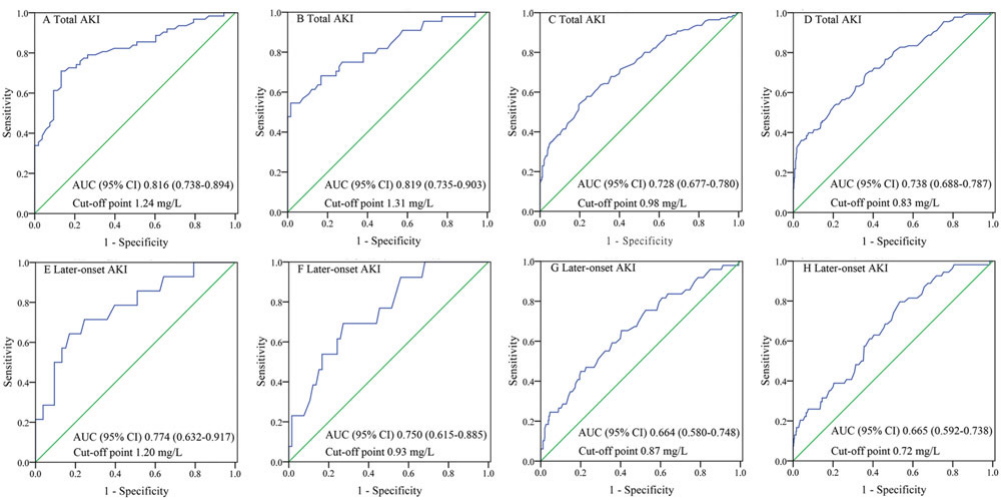
Performance of sCysC for AKI detection after patient stratification by history of diabetes and HbA1c levels. A, recognized diabetes; B, unrecognized diabetes; C, prediabetes; D, normal glycemic status; E, recognized diabetes; F, unrecognized diabetes; G, prediabetes; H, normal glycemic status. The patients with ‘recognized diabetes’ was identified as those with previous diagnosis of diabetes; patients without previous diagnosis of diabetes were further classified according to the level of HbA1c at ICU admission as ‘unrecognized diabetes’ (HbA1c ≥6.5%), ‘prediabetes’ (HbA1c within the range 5.7% to 6.4%), and ‘normal glycemic status’ (HbA1c <5.7%). AKI: acute kidney injury; AUC: area under the curve; CI: confidence interval; Later-onset AKI, defined as no AKI diagnosis at ICU admission but reaching the KDIGO criteria within 1 week after admission; sCysC, serum cystatin C; HbA1c, glycated hemoglobin.

Moreover, the optimal cutoff value of sCysC for detecting total AKI was markedly higher in diabetic patients than in those without diabetes (1.24 mg/L in the group with recognized diabetes, 1.31 mg/L in the group with unrecognized diabetes, 0.98 mg/L in the group with prediabetes, and 0.83 mg/L in the group with normal glycemic status). Similarly, the optimal cutoff value of sCysC for predicting later-onset AKI was calculated as 1.20 mg/L in the group with recognized diabetes, 0.93 mg/L in the group with unrecognized diabetes, 0.87 mg/L in the group with prediabetes, and 0.72 mg/L in the group with normal glycemic status (Table 5 and Figure 2).

In the entire cohort, the patients with diabetes had markedly higher levels of sCysC than those without diabetes (Additional file 6: Supplementary Table S6). Moreover, the incidence of total AKI in the diabetic patients was greater compared to that in those without diabetes (*p* < .001). Similarly, higher incidence of later-onset AKI was noted in patients with diabetes versus those without diabetes (Additional file 7: Supplementary Table S7).

## Discussion

This large prospective study demonstrated that glycemic status had no significant impact on the discriminative ability of sCysC for AKI in critically ill adults and suggested that a higher optimal cutoff value of sCysC for AKI detection should be considered in patients with diabetes.

Serum CysC is a promising and clinical available AKI biomarker worldwide. However, conflicting results [14–16] limit its widespread application for AKI detection. Of note, elevated levels of sCysC are independently associated with HbA1c levels [21], diabetes [17,18], and prediabetes [19,20] other than AKI. Importantly, diabetes is a well-recognized risk factor for AKI [26] and Oezkur et al. [25] found that elevated levels of HbA1c (≥6.0%) were independently associated with an increased risk of AKI. Therefore, it is necessary to demonstrate the comparative performance of sCysC for AKI detection in heterogeneous patient populations with different glycemic status. HbA1c is an established marker to diagnose diabetes and estimate chronic hyperglycemia control. Recent evidence suggests that HbA1c is not affected by critical illness and remains a reliable indicator to estimate previous glycemic control in adult patients admitted to ICU [40]. Compared to the fasting blood glucose, the HbA1c has advantages, such as less day-to-day perturbations during critically illness condition and more convenience [40,41]. The present study was conducted in a general adult ICU with a heterogeneous cohort, and hence it is difficult to obtain fasting blood glucose. Based on these reasons, HbA1s and blood glucose at ICU admission were chosen, instead of fasting blood glucose. To our knowledge, thus far, no study has examined the influence of HbA1c on the discriminatory ability of sCysC for AKI in adult critically ill patients. In the current study, weak correlations between sCysC and HbA1c and between sCysC and history of diabetes were observed. Furthermore, as expected, sCysC was positively associated with HbA1c after adjusting for age, sex, BMI, history of DM, and CKD. On one hand, the levels of sCysC are essentially determined by the GFR [10]. Both diabetes and chronic poor glycemic control could contribute to a higher frequency of renal injury, which was translated into higher concentrations of sCysC [18]. On the other hand, compared to nondiabetic patients, normoalbuminuric diabetic patients without CKD present higher cystatin C levels [18]. Additionally, sCysC levels were found to increase with the increase of HbA1c in diabetic patients without renal injury [42]. It has been reported that insulin resistance is independently increased with increasing HbA1c levels [43], while sCysC levels are associated with insulin resistance regardless of renal function [44]. Accumulating evidence suggest that elevated HbA1c levels may have a role in interfering sCysC concentrations.

However, the difference of AUC among different quartiles of HbA1c or admission serum glucose did not reach statistical significance. Our findings suggest that neither admission HbA1c nor serum glucose have significant impact on the accuracy of sCysC for AKI detection, and therefore, the application of sCysC for AKI detection in heterogeneous patient populations with various glycemic status seems to be appropriate. There were inconsistent results regarding the prognostic value of CysC for kidney function in diabetic patients. In a study of 121 diabetic patients undergoing coronary angiography, Sławomir et al. [45] concluded that sCysC was not more useful than eGFR in the evaluation of kidney function in diabetic patients with coronary heart disease. Nevertheless, Arun et al. [46] suggested that an increase in sCysC levels may indicate acute renal insufficiency in both diabetic and nondiabetic patients after coronary artery bypass grafting surgery procedure. In this large heterogeneous cohort, the accuracy of sCysC in detecting AKI was not compromised in patients with diabetes or prediabetes and supports its clinical applicability in these subpopulations. Interestingly, higher optimal cutoff values of sCysC for AKI detection were recorded in patients with diabetes (both recognized and unrecognized diabetes) versus those without diabetes. The finding of this study indicates that a higher optimal cutoff value of sCysC should be noticed in critically ill adults with diabetes for AKI detection. The decrease of renal function is the most important factor for the increment of sCysC from baseline, thereby the baseline renal function may play an important part in the higher optimal cutoff value of sCysC in diabetic patients. In our study, patients with diabetes or prediabetes demonstrated higher levels of serum creatinine and rates of CKD in comparison to their counterparts without diabetes or with lower HbA1c levels. Both diabetes and chronic poor glycemic control (high HbA1c levels) could contribute to a higher frequency of diabetic nephropathy, which was translated into higher concentrations of sCysC [18]. Accordingly, this is the potential reason for different sCysC cutoff values in these subgroups.

Our study has several limitations. First, we measured sCysC only once at ICU admission. As the Acute Dialysis Quality Initiative (ADQI) cannot recommend a serial testing schedule [26], it is not practical and cost-effective for collecting and measuring a series of samples at frequent time points. We speculate that our conclusions are not debilitated by this limitation. Second, sCysC levels could theoretically be elevated due to a variety of conditions, such as inflammation or thyroid function, and not specifically in response to AKI [10,47,48]. These factors such as thyroid function and C-reactive protein (CRP) were not included in this study. Although prior reports [47,48] found that thyroid function, CRP, or BMI did not have statistically significant influence on the diagnostic accuracy of sCysC for AKI detection, further study and discussion is required. Third, in our population, serum creatinine at ICU admission was selected for baseline creatinine only when pre-ICU baseline creatinine was not available [31]. Although serum creatinine at ICU admission is the last preference for baseline creatinine selection, the problem of steady state of serum creatinine at ICU admission should be considered. Fourth, we did not consider the standardization of potential confounders related to sCysC prior to ROC analysis in each HbA1c quartiles. Regarding to potential confounders, future study is need. Considering that HbA1c is an easy-to-use and reliable laboratory marker, we mainly focus on its effect on the discriminatory ability of sCysC for AKI. In the present study, sCysC could detect AKI with reasonable certainty in 4 quartiles based on HbA1c levels. Thus, we use similar statistical method referring to the previous study about the impacts of thyroid function on the diagnostic accuracy of sCysC for AKI [48]. Last but not least, low APACHE II scores were observed in our cohort, which may limited the generalizability of our conclusion.

## Conclusion

The present study indicates that glycemic status has no significant impact on the performance of sCysC to detect AKI and a higher optimal cutoff value of sCysC should be considered for AKI detection in critically ill adults with diabetes. As this study was conducted in a large population of ICU patients, our findings could have useful clinical implications for actual heterogeneous critically ill adults at risk for AKI.

## Supplementary Material

Supplementary Table 7

Supplementary Table 6

Supplementary Table 5

Supplementary Table 4

Supplementary Table 3

Supplementary Table 2

Supplementary Table 1

## Abbreviation

ADQI: the acute dialysis quality initiative
AKI: acute kidney injury
APACHE II: Acute Physiology and Chronic Health Evaluation score
AUC-ROC: area under the receiver operating characteristic curve
BMI: body mass index
CAD: coronary artery disease
CI: confidence interval
CKD: chronic kidney disease, defined as baseline eGFR < 60 ml/min per 1.73m^2^
CRP: C-reactive protein
DM: diabetes mellitus
eGFR: estimated glomerular filtration rate
ESRD: end-stage renal disease
HbA1c: glycated hemoglobin
HPLC: high-performance liquid chromatography
ICU: intensive care unit
KDIGO: kidney disease improving global outcomes criteria
MDRD: modification of diet in renal disease formula
RRT: renal replacement therapy
sCr: serum creatinine
sCysC: serum cystatin C.

## References

[1] Xu X, Nie S, Liu Z, et al. Epidemiology and clinical correlates of AKI in Chinese hospitalized adults. Clin J Am Soc Nephrol. 2015;10:1510–1518. 26231194 10.2215/CJN.02140215 PMC4559507

[2] Lameire NH, Bagga A, Cruz D, et al. Acute kidney injury: an increasing global concern. Lancet. 2013;382:170–179. 23727171 10.1016/S0140-6736(13)60647-9

[3] Bihorac A, Chawla LS, Shaw AD, et al. Validation of cell-cycle arrest biomarkers for acute kidney injury using clinical adjudication. Am J Respir Crit Care Med. 2014;189:932–939. 24559465 10.1164/rccm.201401-0077OC

[4] de Geus HR, Bakker J, Lesaffre EM, et al. Neutrophil gelatinase-associated lipocalin at ICU admission predicts for acute kidney injury in adult patients. Am J Respir Crit Care Med. 2011;183:907–914. 20935115 10.1164/rccm.200908-1214OC

[5] Han WK, Bailly V, Abichandani R, et al. Kidney injury molecule-1 (KIM-1): a novel biomarker for human renal proximal tubule injury. Kidney Int. 2002;62:237–244. 12081583 10.1046/j.1523-1755.2002.00433.x

[6] Deng Y, Chi R, Chen S, et al. Evaluation of clinically available renal biomarkers in critically ill adults: a prospective multicenter observational study. Crit Care. 2017;21:46. 28264714 10.1186/s13054-017-1626-0 PMC5339963

[7] Deng Y, Yuan J, Chi R, et al. The incidence, risk factors and outcomes of postoperative acute kidney injury in neurosurgical critically ill patients. Sci Rep. 2017;7:4245. 28652590 10.1038/s41598-017-04627-3 PMC5484679

[8] Zhang D, Gao L, Ye H, et al. Impact of thyroid function on cystatin C in detecting acute kidney injury: a prospective, observational study. BMC Nephrol. 2019;20:41. 30727972 10.1186/s12882-019-1201-9 PMC6364411

[9] Kreepala C, Kitporntheranunt M, Sangwipasnapaporn W, et al. Assessment of preeclampsia risk by use of serum ionized magnesium-based equation. Ren Fail. 2018;40:99–106. 29318926 10.1080/0886022X.2017.1422518 PMC6014514

[10] Charlton JR, Portilla D, Okusa MD A basic science view of acute kidney injury biomarkers. Nephrol Dial Transplant. 2014;29:1301–1311. 24385545 10.1093/ndt/gft510 PMC4081632

[11] Bell M, Granath F, Martensson J, et al. Cystatin C is correlated with mortality in patients with and without acute kidney injury. Nephrol Dial Transplant. 2009;24:3096–3102. 19395727 10.1093/ndt/gfp196

[12] Ostermann M, Joannidis M Acute kidney injury 2016: diagnosis and diagnostic workup. Crit Care. 2016;20:299 27670788 10.1186/s13054-016-1478-z PMC5037640

[13] Kreepala C, Luangphiphat W, Villarroel A, et al. Effect of magnesium on glomerular filtration rate and recovery of hypertension in women with severe preeclampsia. Nephron. 2018;138:35–41. 29176311 10.1159/000481463

[14] Nejat M, Pickering JW, Walker RJ, et al. Rapid detection of acute kidney injury by plasma cystatin C in the intensive care unit. Nephrol Dial Transplant. 2010;25:3283–3289. 20350927 10.1093/ndt/gfq176

[15] Soto K, Coelho S, Rodrigues B, et al. Cystatin C as a marker of acute kidney injury in the emergency department. Clin J Am Soc Nephrol. 2010;5:1745–1754. 20576828 10.2215/CJN.00690110 PMC2974372

[16] Royakkers AA, Korevaar JC, van Suijlen JD, et al Serum and urine cystatin C are poor biomarkers for acute kidney injury and renal replacement therapy. Intensive Care Med. 2011;37:493–501. 21153403 10.1007/s00134-010-2087-y PMC3042095

[17] Yashiro M, Kamata T, Segawa H, et al. Comparisons of cystatin C with creatinine for evaluation of renal function in chronic kidney disease. Clin Exp Nephrol. 2009;13:598–604. 19585181 10.1007/s10157-009-0202-6

[18] Borges RL, Hirota AH, Quinto BM, et al. Is cystatin C a useful marker in the detection of diabetic kidney disease? Nephron Clin Pract. 2010;114:c127–c134. 19887833 10.1159/000254385

[19] Donahue RP, Stranges S, Rejman K, et al. Elevated cystatin C concentration and progression to pre-diabetes: the Western New York study. Diabetes Care. 2007;30:1724–1729. 17456840 10.2337/dc07-0040

[20] Sabanayagam C, Wong TY, Xiao J, et al. Serum cystatin C and prediabetes in non-obese US adults. Eur J Epidemiol. 2013;28:311–316. 23417728 10.1007/s10654-013-9781-3

[21] Isobe S, Yamada T, Yuba M, et al. Relationship between pre-procedural microalbuminuria and renal functional changes after coronary computed tomography in diabetic patients. J Cardiol. 2017;69:666–760. 27424108 10.1016/j.jjcc.2016.06.005

[22] Carpenter DL, Gregg SR, Xu K, et al. Prevalence and Impact of Unknown Diabetes in the ICU. Crit Care Med. 2015;43:e541–e550. 26465219 10.1097/CCM.0000000000001353

[23] Plummer MP, Bellomo R, Cousins CE, et al. Dysglycaemia in the critically ill and the interaction of chronic and acute glycaemia with mortality. Intensive Care Med. 2014;40:973–980. 24760120 10.1007/s00134-014-3287-7

[24] Kompoti M, Michalia M, Salma V, et al. Glycated hemoglobin at admission in the intensive care unit: clinical implications and prognostic relevance. J Crit Care. 2015;30:150–155. 25239822 10.1016/j.jcrc.2014.08.014

[25] Oezkur M, Wagner M, Weismann D, et al. Chronic hyperglycemia is associated with acute kidney injury in patients undergoing CABG surgery–a cohort study. BMC Cardiovasc Disord. 2015;15:41. 25964053 10.1186/s12872-015-0028-y PMC4443518

[26] McCullough PA, Shaw AD, Haase M, et al. Diagnosis of acute kidney injury using functional and injury biomarkers: workgroup statements from the tenth Acute Dialysis Quality Initiative Consensus Conference. Contrib Nephrol. 2013;182:13–29. 23689653 10.1159/000349963

[27] von Elm E, Altman DG, Egger M, et al. The Strengthening the Reporting of Observational Studies in Epidemiology (STROBE) statement: guidelines for reporting observational studies. Ann Intern Med. 2007;147:573–577. 17938396 10.7326/0003-4819-147-8-200710160-00010

[28] Bossuyt PM, Reitsma JB, Bruns DE, et al. Toward complete and accurate reporting of studies of diagnostic accuracy: the STARD initiative. Acad Radiol. 2003;10:664–669. 12809421 10.1016/s1076-6332(03)80086-7

[29] Levey AS, Coresh J, Greene T, et al. Expressing the Modification of Diet in Renal Disease Study equation for estimating glomerular filtration rate with standardized serum creatinine values. Clin Chem. 2007;53:766–772. 17332152 10.1373/clinchem.2006.077180

[30] Kidney Disease Improving Global Outcomes (KDIGO) Acute Kidney Injury Work Group: KDIGO clinical practice guideline for acute kidney injury. Kidney Int Suppl. 2012;2:1–138.

[31] Endre ZH, Walker RJ, Pickering JW, et al. Early intervention with erythropoietin does not affect the outcome of acute kidney injury (the EARLYARF trial). Kidney Int. 2010;77:1020–1030. 20164823 10.1038/ki.2010.25

[32] Bone RC, Balk RA, Cerra FB, et al. Definitions for sepsis and organ failure and guidelines for the use of innovative therapies in sepsis. The ACCP/SCCM Consensus Conference Committee. American College of Chest Physicians/Society of Critical Care Medicine. Chest. 1992;101:1644–1655. 1303622 10.1378/chest.101.6.1644

[33] Zhang Z, Lu B, Sheng X, et al. Cystatin C in prediction of acute kidney injury: a systemic review and meta-analysis. Am J Kidney Dis. 2011;58:356–365. 21601330 10.1053/j.ajkd.2011.02.389

[34] Filler G, Bokenkamp A, Hofmann W, et al Cystatin C as a marker of GFR-history, indications, and future research. Clin Biochem. 2005;38:1–8. 15607309 10.1016/j.clinbiochem.2004.09.025

[35] American Diabetes Association. Diagnosis and classification of diabetes mellitus. Diabetes Care. 2013;36: S67–S74. 23264425 10.2337/dc13-S067 PMC3537273

[36] Hanley JA, McNeil BJ The meaning and use of the area under a receiver operating characteristic (ROC) curve. Radiology. 1982;143:29–36. 7063747 10.1148/radiology.143.1.7063747

[37] Hanley JA, McNeil BJ A method of comparing the areas under receiver operating characteristic curves derived from the same cases. Radiology. 1983;148:839–843. 6878708 10.1148/radiology.148.3.6878708

[38] Glassford NJ, Schneider AG, Xu S, et al. The nature and discriminatory value of urinary neutrophil gelatinase-associated lipocalin in critically ill patients at risk of acute kidney injury. Intensive Care Med. 2013;39:1714–1724. 23917325 10.1007/s00134-013-3040-7

[39] Youden WJ Index for rating diagnostic tests. Cancer. 1950;3:32–35. 15405679 10.1002/1097-0142(1950)3:1<32::aid-cncr2820030106>3.0.co;2-3

[40] Luethi N, Cioccari L, Tanaka A, et al. Glycated Hemoglobin A1c Levels Are Not Affected by Critical Illness. Crit Care Med. 2016;44:1692–1694. 26977855 10.1097/CCM.0000000000001656

[41] Erratum Classification and diagnosis of diabetes. Sec. 2. In Standards of Medical Care in Diabetes-2016. Diabetes Care. 2016;39:1653. 27555625 10.2337/dc16-er09

[42] Qian T, Tian L, Li Y, et al. Value of the combined examination of Cys-C and HbA1c for diagnosis of early renal injury in pediatric diabetes. Exp Ther Med. 2017;13:515–518. 28352324 10.3892/etm.2016.3967 PMC5348714

[43] Hou X, Liu J, Song J, et al. Relationship of hemoglobin A1c with beta cell function and insulin resistance in newly diagnosed and drug naive type 2 diabetes patients. J Diab Res. 2016;2016:1. 10.1155/2016/8797316 PMC4657079 26640807

[44] Lee SH, Park SA, Ko SH, et al. Insulin resistance and inflammation may have an additional role in the link between cystatin C and cardiovascular disease in type 2 diabetes mellitus patients. Metabolism Clin Exp. 2010;59:241–246. 10.1016/j.metabol.2009.07.019 19765773

[45] Matys U, Bachorzewska-Gajewska H, Malyszko J, et al. Assessment of kidney function in diabetic patients. Is there a role for new biomarkers NGAL, cystatin C and KIM-1? Adv Med Sci. 2013;58:353–361. 24384771 10.2478/v10039-012-0077-8

[46] Arun O, Celik G, Oc B, et al. Renal effects of coronary artery bypass graft surgery in diabetic and non-diabetic patients: a study with urinary neutrophil gelatinase-associated lipocalin and serum cystatin C. Kidney Blood Press Res. 2015;40:141–152. 25832128 10.1159/000368490

[47] Schanz M, Pannes D, Dippon J, et al. The Influence of Thyroid Function, Inflammation, and Obesity on Risk Prediction of Acute Kidney Injury by Cystatin C in the Emergency Department. Kidney Blood Press Res. 2016;41:604–613. 27578194 10.1159/000447929

[48] Wang F, Pan W, Wang H, et al. The impacts of thyroid function on the diagnostic accuracy of cystatin C to detect acute kidney injury in ICU patients: a prospective, observational study. Crit Care. 2014;18:R9. 24405645 10.1186/cc13186 PMC4056557

