# Supplementary material for: The influence of glycemic status on the performance of cystatin C for acute kidney injury detection in the critically ill
Source: Ren Fail. 2019 Apr 3;41(1):139–49. doi: 10.1080/0886022X.2019.1586722 (PMC6450510; doi:10.1080/0886022X.2019.1586722)
Supplement: Supplementary Table 7 [file IRNF_A_1586722_SM8486.docx]

**Supplementary Table 7.** Characteristics of participants without established AKI according to HbA1c levels and history of diabetes

|  |  | Patients without previous diagnosis of diabetes | | | |
| --- | --- | --- | --- | --- | --- |
| Variables | Recognised  diabetes | Unrecognised diabetes | Prediabetes | Normal glycaemic status | *P* |
| Number | 67 | 79 | 393 | 529 | / |
| Age, years | 65 (57-76)**^a^** | 60 (54-66)**^b^** | 57 (47-66)**^b^** | 47 (35-58) | <0.001 |
| Males, n (%) | 38 (56.7) | 43 (54.4) | 199 (50.6) | 274 (51.8) | 0.782 |
| BMI, kg/m^2^ | 22.32 (21.47-25.69) | 22.72 (21.87-27.34)**^c^** | 22.43(21.16-24.50)**^b^** | 22.19 (20.72-23.07) | <0.001 |
| Later-onset AKI, n (%) | 14 (20.9) | 13 (16.5) | 49 (12.5) | 54 (10.2) | 0.045 |
| CKD, n (%) | 5 (7.5) | 3 (3.8) | 18 (4.6) | 12 (2.3) | 0.080 |
| APACHE II | 12 (9-17)**^c^** | 12 (9-17)**^b^** | 11 (8-15)**^b^** | 9 (7-13) | <0.001 |
| sCr at ICU admission, mg/dL | 0.87 (0.67-1.02) | 0.79 (0.62-0.93) | 0.80 (0.68-0.98) | 0.77 (0.64-0.92) | 0.017 |
| sCysC at ICU admission, mg/L | 0.95 (0.71-1.20)**^c^** | 0.84 (0.68-1.02)**^b^** | 0.83 (0.66-1.01)**^b^** | 0.74 (0.61-0.94) | <0.001 |
| Serum glucose at ICU admission, mg/dL | 177.3(132.3-238.9)**^a^** | 148.0(123.5-187.4)**^c^** | 122.9(103.8-147.2)**^b^** | 117.7 (101.9-138.0) | <0.001 |
| HbA1c at ICU admission, % | 7.2 (6.5-8.1)**^a^** | 6.7 (6.6-7.4)**^d^** | 5.9 (5.8-6.1)**^e^** | 5.3 (5.1-5.5)**^f^** | <0.001 |

**Abbreviation: HbA1c, glycosylated haemoglobin; established AKI, diagnosis of AKI at ICU admission; BMI, Body mass index; Later-onset AKI, indicated no AKI diagnosis at ICU admission but reaching the KDIGO criteria within 1 week after admission; CKD, chronic kidney disease, defined as baseline eGFR <60 mL/min/1.73 m^2^; eGFR, estimated glomerular ﬁltration rate; APACHE II, Acute Physiology and Chronic Health Evaluation score; sCr, serum creatinine; ICU, intensive care unit; sCysC, serum cystatin C.**

**The non-normally distributed continuous variables are expressed as median (25th percentile to 75th percentile [interquartile range]). Categorical variables are expressed as n (%).**

**Patients without established AKI** **were stratified into 4 groups according to HbA1c levels and history of diabetes: recognised diabetes, unrecognised diabetes, prediabetes, and normal glycaemic status.**

**The ‘recognised diabetes’ was identified using the hospital case records and history provided by patients or their family; patients without previous diagnosis of diabetes were further classified according to the level of HbA1c at ICU admission as ‘unrecognised diabetes’ (HbA1c ≥6.5%), ‘prediabetes’ (HbA1c within the range 5.7% to 6.4%), and ‘normal glycaemic status’ (HbA1c < 5.7%).**

**Group I: recognised diabetes; Group II: unrecognised diabetes; Group III: prediabetes; Group IV: normal glycaemic status.**

**^a^*P*<0.05 vs. Group II, Group III, and Group IV; ^b^*P*<0.05 vs. Group IV; and ^c^*P*<0.05 vs. Group III and Group IV; ^d^*P*<0.05 vs. Group I, Group III, and Group IV; ^e^*P*<0.05 vs. Group I, Group II and Group IV; ^f^*P*<0.05 vs. Group I, Group II, and Group III.**
