# Supplementary material for: The influence of glycemic status on the performance of cystatin C for acute kidney injury detection in the critically ill
Source: Ren Fail. 2019 Apr 3;41(1):139–49. doi: 10.1080/0886022X.2019.1586722 (PMC6450510; doi:10.1080/0886022X.2019.1586722)
Supplement: Supplementary Table 6 [file IRNF_A_1586722_SM8485.docx]

**Supplementary Table 6.** Characteristics of enrolled patients according to HbA1c levels and history of diabetes

|  |  | Patients without previous diagnosis of diabetes | | | |
| --- | --- | --- | --- | --- | --- |
| Variables | Recognised  diabetes | Unrecognised  diabetes | Prediabetes | Normal glycaemic  status | *P* |
| Number | 115 | 110 | 484 | 608 | / |
| Age, years | 66 (58-74)**^a^** | 62 (55-68)**^b^** | 57 (47-66)**^c^** | 48 (35-59)**^d^** | <0.001 |
| Males, n (%) | 66 (57.4) | 68 (61.8) | 258 (53.3) | 329 (54.1) | 0.384 |
| BMI, kg/m^2^ | 22.32 (21.50-24.56)**^e^** | 22.72(21.90-27.05)**^e^** | 22.46(21.40-24.34)**^e^** | 22.19 (20.83-23.07) | <0.001 |
| Total AKI, n (%) | 62 (53.9) | 44 (40.0) | 140 (28.9) | 133 (21.9) | <0.001 |
| CKD, n (%) | 23 (20.0) | 9 (8.2) | 30 (6.2) | 25 (4.1) | <0.001 |
| APACHE II | 16 (11-24)**^f^** | 14 (10-22)**^f^** | 11 (8-17)**^e^** | 10 (7-14) | <0.001 |
| sCr at ICU admission, mg/dL | 1.01 (0.79-1.36)**^a^** | 0.89 (0.67-1.07) | 0.85 (0.70-1.07)**^e^** | 0.80 (0.66-0.98) | <0.001 |
| sCysC at ICU admission, mg/L | 1.10 (0.83-1.64)**^a^** | 0.90 (0.75-1.24)**^e^** | 0.86 (0.69-1.11)**^e^** | 0.77 (0.62-1.00) | <0.001 |
| Serum glucose at ICU admission, mg/dL | 188.6(142.7-243.5)**^a^** | 149.0(123.8-203.5)**^b^** | 126.8(105.2-152.1)**^c^** | 119.2 (103.0-140.2)**^d^** | <0.001 |
| HbA1c at ICU admission, % | 7.1 (6.4-8.2)**^f^** | 6.8 (6.6-7.4)**^f^** | 5.9 (5.8-6.1)**^e^** | 5.3 (5.1-5.5) | <0.001 |

**Abbreviation: HbA1c, glycosylated haemoglobin; BMI, Body mass index; CKD, chronic kidney disease, defined as baseline eGFR <60 mL/min/1.73 m^2^; eGFR, estimated glomerular ﬁltration rate; APACHE II, Acute Physiology and Chronic Health Evaluation score; sCr, serum creatinine; ICU, intensive care unit; sCysC, serum cystatin C.**

**The non-normally distributed continuous variables are expressed as median (25th percentile to 75th percentile [interquartile range]). Categorical variables are expressed as n (%).**

**Patients were stratified into 4 groups according to HbA1c levels and history of diabetes: recognised diabetes, unrecognised diabetes, prediabetes, and normal glycaemic status.**

**The ‘recognised diabetes’ was identified using the hospital case records and history provided by patients or their family; patients without previous diagnosis of diabetes were further classified according to the level of HbA1c at ICU admission as ‘unrecognised diabetes’ (HbA1c ≥6.5%), ‘prediabetes’ (HbA1c within the range 5.7% to 6.4%), and ‘normal glycaemic status’ (HbA1c < 5.7%).**

**Group I: recognised diabetes; Group II: unrecognised diabetes; Group III: prediabetes; Group IV: normal glycaemic status.**

**^a^*P* <0.05 vs. Group II, Group III, and Group IV; ^b^*P* <0.05 vs. Group I, Group III, and Group IV; ^c^*P* <0.05 vs. Group I, Group II, and Group IV; ^d^ *P*<0.05 vs. Group I, Group II, and Group III; ^e^*P* <0.05 vs. Group IV; ^f^*P* <0.05 vs. Group III and Group IV.**
