# Supplementary material for: The influence of glycemic status on the performance of cystatin C for acute kidney injury detection in the critically ill
Source: Ren Fail. 2019 Apr 3;41(1):139–49. doi: 10.1080/0886022X.2019.1586722 (PMC6450510; doi:10.1080/0886022X.2019.1586722)
Supplement: Supplementary Table 5 [file IRNF_A_1586722_SM8484.docx]

**Supplementary Table 5.** Characteristics of participants without established AKI according to quartiles of serum glucose

| Variables | Quartile I | Quartile II | Quartile III | Quartile IV | *P* |
| --- | --- | --- | --- | --- | --- |
| Number | 265 | 265 | 270 | 268 | / |
| Age, years | 51 (39-63)**^a^** | 51 (38-60)**^a^** | 53 (41-63)**^a^** | 59 (48-68) | <0.001 |
| Males, n (%) | 148 (55.8) | 148 (55.8) | 127 (47.0) | 129 (48.1) | 0.061 |
| BMI, kg/m^2^ | 22.46 (20.81-24.28)**^b^** | 21.93 (19.95-23.09)**^c^** | 22.19 (21.76-23.46) | 22.32 (21.41-25.45) | <0.001 |
| Later-onset AKI, n (%) | 21 (7.9) | 25 (9.4) | 33 (12.2) | 51 (19.0) | <0.001 |
| History of diabetes, n (%) | 3 (1.1) | 11 (4.2) | 7 (2.6) | 46 (17.2) | <0.001 |
| CKD, n (%) | 6 (2.3) | 9 (3.4) | 11 (4.1) | 12 (4.5) | 0.535 |
| APACHE II | 10 (7-13)**^c^** | 9 (7-12)**^c^** | 10 (8-14)**^a^** | 12 (9-18) | <0.001 |
| sCr at ICU admission, mg/dL | 0.80 (0.66-0.96) | 0.80 (0.66-0.96) | 0.78 (0.66-0.95) | 0.78 (0.67-0.92) | 0.903 |
| sCysC at ICU admission, mg/L | 0.88 (0.71-1.09)**^d^** | 0.80 (0.63-0.97) | 0.75 (0.63-0.92) | 0.78 (0.61-0.98) | <0.001 |
| Serum glucose at ICU admission, mg/dL | 94.7 (89.2-99.9)**^d^** | 113.2 (107.9-118.2)**^e^** | 133.9 (127.4-140.2)**^f^** | 173.2 (157.6-207.6)**^g^** | <0.001 |
| HbA1c at ICU admission, % | 5.5 (5.2-5.9)**^c^** | 5.6 (5.3-5.8)**^c^** | 5.7 (5.3-6.0)**^a^** | 5.9 (5.5-6.6) | <0.001 |

**Abbreviation: AKI, acute kidney injury; established AKI, diagnosis of AKI at ICU admission; BMI, Body mass index; Later-onset AKI, indicated no AKI diagnosis at ICU admission but reaching the KDIGO criteria within 1 week after admission; ICU, intensive care unit; CKD, chronic kidney disease, defined as baseline eGFR <60 mL/min/1.73 m^2^; eGFR, estimated glomerular ﬁltration rate; APACHE II, Acute Physiology and Chronic Health Evaluation score; sCr, serum creatinine; sCysC, serum cystatin C; HbA1c, glycosylated haemoglobin.**

**The non-normally distributed continuous variables are expressed as median (25th percentile to 75th percentile [interquartile range]). Categorical variables are expressed as n (%).**

**Patients without established AKI were stratified into 4 quartiles according to serum glucose at ICU admission. Quartile cut points for serum glucose at ICU admission were 104.3 mg/dl, 121.9 mg/dl, and 148.5 mg/dl.**

**^a^*P* <0.05 vs. Quartile IV; ^b^*P* <0.05 vs. Quartile II; ^c^*P* <0.05 vs. Quartile III, and Quartile IV; ^d^*P* <0.05 vs. Quartile II, Quartile III, and Quartile IV; ^e^ *P*<0.05 vs. Quartile I, Quartile III, and Quartile IV; ^f^*P* <0.05 vs. Quartile I, Quartile II, and Quartile IV; ^g^*P* <0.05 vs. Quartile I, Quartile II, and Quartile III.**
