# Supplementary material for: The influence of glycemic status on the performance of cystatin C for acute kidney injury detection in the critically ill
Source: Ren Fail. 2019 Apr 3;41(1):139–49. doi: 10.1080/0886022X.2019.1586722 (PMC6450510; doi:10.1080/0886022X.2019.1586722)
Supplement: Supplementary Table 4 [file IRNF_A_1586722_SM8483.docx]

**Supplementary Table 4.** Characteristics of enrolled patients according to quartiles of serum glucose

| Variables | Quartile I | Quartile II | Quartile III | Quartile IV | *P* |
| --- | --- | --- | --- | --- | --- |
| Number | 328 | 329 | 331 | 329 | / |
| Age, years | 51 (39-63)**^a^** | 52 (39-62)**^b^** | 55 (43-65)**^b^** | 61 (50-70) | <0.001 |
| Males, n (%) | 185 (56.4) | 192 (58.4) | 169 (51.1) | 176 (53.5) | 0.249 |
| BMI, kg/m^2^ | 22.46 (20.88-24.11) | 22.06 (20.70-23.26)**^a^** | 22.20 (21.55-23.89) | 22.23 (21.43-24.46) | 0.005 |
| Total AKI, n (%) | 52 (15.9) | 73 (22.2) | 102 (30.8) | 152 (46.2) | <0.001 |
| History of diabetes, n (%) | 6 (1.8) | 12 (3.6) | 18 (5.4) | 79 (24.0) | <0.001 |
| CKD, n (%) | 9 (2.7) | 15 (4.6) | 25 (7.6) | 38 (11.6) | <0.001 |
| APACHE II | 10 (7-13)**^a^** | 10 (7-13)**^a^** | 11 (8-17)**^b^** | 15 (10-23) | <0.001 |
| sCr at ICU admission, mg/dL | 0.84 (0.68-0.98)**^b^** | 0.83 (0.67-1.01)**^b^** | 0.83 (0.68-1.06) | 0.88 (0.70-1.18) | 0.001 |
| sCysC at ICU admission, mg/L | 0.89 (0.72-1.11)**^c^** | 0.81 (0.64-1.00)**^b^** | 0.79 (0.65-1.04)**^b^** | 0.87 (0.66-1.27) | <0.001 |
| Serum glucose at ICU admission, mg/dL | 95.6 (89.6-101.5)**^d^** | 116.3 (111.5-20.7)**^e^** | 139.3 (133.2-146.2)**^f^** | 185.9 (166.0-22.9)**^g^** | <0.001 |
| HbA1c at ICU admission, % | 5.5 (5.3-5.9)**^a^** | 5.6 (5.3-5.9)**^a^** | 5.7 (5.4-6.1)**^b^** | 6.0 (5.6-6.8) | <0.001 |

**Abbreviation: ICU, intensive care unit; BMI, Body mass index; AKI, acute kidney injury; CKD, chronic kidney disease, defined as baseline eGFR <60 mL/min/1.73 m^2^; eGFR, estimated glomerular ﬁltration rate; APACHE II, Acute Physiology and Chronic Health Evaluation score; sCr, serum creatinine; sCysC, serum cystatin C; HbA1c, glycosylated haemoglobin.**

**The non-normally distributed continuous variables are expressed as median (25th percentile to 75th percentile [interquartile range]). Categorical variables are expressed as n (%).**

**Patients were stratified into 4 quartiles according to serum glucose at ICU admission.**

**Quartile cut points for serum glucose at ICU admission were 106.0 mg/dl, 126.5 mg/dl, and 154.5 mg/dl.**

**^a^*P* <0.05 vs. Quartile III, and Quartile IV; ^b^*P* <0.05 vs. Quartile IV; ^c^*P* <0.05 vs. Quartile II and Quartile III; ^d^*P*<0.05 vs. Quartile II, Quartile III, and Quartile IV; ^e^*P* <0.05 vs. Quartile I, Quartile III, and Quartile IV; ^f^*P* <0.05 vs. Quartile I, Quartile II, and Quartile IV; ^g^*P* <0.05 vs. Quartile I, Quartile II, and Quartile III.**
