# Supplementary material for: The influence of glycemic status on the performance of cystatin C for acute kidney injury detection in the critically ill
Source: Ren Fail. 2019 Apr 3;41(1):139–49. doi: 10.1080/0886022X.2019.1586722 (PMC6450510; doi:10.1080/0886022X.2019.1586722)
Supplement: Supplementary Table 3 [file IRNF_A_1586722_SM8482.docx]

**Supplementary Table 3.** Performance of sCysC in detecting AKI according to quartiles of serum glucose

| Group | AUC-ROC | 95% CI | *P* | Cut-off (mg/L) | SENS | SPEC |
| --- | --- | --- | --- | --- | --- | --- |
| Total AKI |  |  |  |  |  |  |
| Quartile I | 0.720 ± 0.039 | 0.645-0.796 | < 0.001 | 1.32 | 0.404 | 0.960 |
| Quartile II | 0.755 ± 0.035 | 0.686-0.824 | < 0.001 | 0.97 | 0.630 | 0.805 |
| Quartile III | 0.779 ± 0.030 | 0.721-0.837 | < 0.001 | 1.03 | 0.559 | 0.878 |
| Quartile IV | 0.804 ± 0.024 | 0.757-0.851 | < 0.001 | 1.04 | 0.625 | 0.853 |
| Later-onset AKI |  |  |  |  |  |  |
| Quartile I | 0.647 ± 0.062 | 0.525-0.769 | 0.026 | 0.72 | 1.000 | 0.275 |
| Quartile II | 0.742 ± 0.059 | 0.626-0.858 | < 0.001 | 0.97 | 0.680 | 0.796 |
| Quartile III | 0.648 ± 0.049 | 0.551-0.745 | 0.006 | 0.73 | 0.758 | 0.498 |
| Quartile IV | 0.744 ± 0.038 | 0.669-0.818 | < 0.001 | 0.92 | 0.608 | 0.774 |

**Abbreviation: sCysC, serum cystatin C; AKI, acute kidney injury; AUC-ROC, area under the receiver operating characteristic curve; CI, confidence interval; SENS, Sensitivity; SPEC, Specificity; Later-onset AKI, indicated no AKI diagnosis at ICU admission but reaching the KDIGO criteria within 1 week after admission.**

**Patients were stratified into 4 quartiles according to serum glucose at ICU admission.**

**Total AKI: quartile cut points for admission serum glucose were 106.0 mg/dl, 126.5 mg/dl, and 154.5 mg/dl.**

**AUC of Quartile I versus AUC of Quartile II, Z = 0.668, *P* = 0.504;**

**AUC of Quartile I versus AUC of Quartile III, Z = 1.199, *P* = 0.231;**

**AUC of Quartile I versus AUC of Quartile IV, Z = 1.834, *P* = 0.067;**

**AUC of Quartile II versus AUC of Quartile III, Z = 0.521, *P* = 0.603;**

**AUC of Quartile II versus AUC of Quartile IV, Z = 1.155, *P* = 0.248;**

**AUC of Quartile III versus AUC of Quartile IV, Z = 0.651, *P* = 0.515.**

**Later-onset AKI: quartile cut points for admission serum glucose were 104.3 mg/dl, 121.9 mg/dl, and 148.5 mg/dl.**

**AUC of Quartile I versus AUC of Quartile II, Z = 1.110, *P* = 0.267;**

**AUC of Quartile I versus AUC of Quartile III, Z = 0.013, *P* = 0.990;**

**AUC of Quartile I versus AUC of Quartile IV, Z = 1.334, *P* = 0.182;**

**AUC of Quartile II versus AUC of Quartile III, Z = 1.226, *P* = 0.220;**

**AUC of Quartile II versus AUC of Quartile IV, Z = 0.029, *P* = 0.977;**

**AUC of Quartile III versus AUC of Quartile IV, Z = 1.548, *P* = 0.122.**
