# Supplementary material for: The influence of glycemic status on the performance of cystatin C for acute kidney injury detection in the critically ill
Source: Ren Fail. 2019 Apr 3;41(1):139–49. doi: 10.1080/0886022X.2019.1586722 (PMC6450510; doi:10.1080/0886022X.2019.1586722)
Supplement: Supplementary Table 2 [file IRNF_A_1586722_SM8481.docx]

**Supplementary Table 2.** Performance of sCysC in detecting AKI

| Outcomes | AUC-ROC | 95% CI | *P* | Cut-off (mg/L) | SENS | SPEC |
| --- | --- | --- | --- | --- | --- | --- |
| Total AKI | 0.765 ± 0.015 | 0.736-0.795 | < 0.001 | 1.04 | 0.567 | 0.826 |
| Later-onset AKI | 0.689 ± 0.025 | 0.641-0.738 | < 0.001 | 1.04 | 0.431 | 0.826 |

**Abbreviation: sCysC, serum cystatin C; AKI, acute kidney injury; Later-onset AKI, indicated no AKI diagnosis at ICU admission but reaching the KDIGO criteria within 1 week after admission; AUC-ROC, area under the receiver operating characteristic curve; CI, confidence interval; SENS, sensitivity; SPEC, specificity.**
