# Supplementary material for: The influence of glycemic status on the performance of cystatin C for acute kidney injury detection in the critically ill
Source: Ren Fail. 2019 Apr 3;41(1):139–49. doi: 10.1080/0886022X.2019.1586722 (PMC6450510; doi:10.1080/0886022X.2019.1586722)
Supplement: Supplementary Table 1 [file IRNF_A_1586722_SM8480.docx]

**Supplementary Table 1.** Baseline characteristics and outcomes

| Characteristics | Non-AKI (n = 938) | AKI (n = 379 ) | *P* |
| --- | --- | --- | --- |
| Demographic variables |  |  |  |
| Age, years | 52 (41-62) | 62 (48-72) | < 0.001 |
| Males, n (%) | 480 (51.2) | 240 (63.3) | < 0.001 |
| BMI, kg/m^2^ | 22.2 (20.9-23.8) | 22.6 (21.4-23.9) | 0.028 |
| Smokers, n (%) | 65 (6.9) | 41 (10.8) | 0.019 |
| Hypertension, n (%) | 145 (15.5) | 138 (36.4) | < 0.001 |
| DM, n (%) | 119 (12.7) | 106 (28.0) | < 0.001 |
| CKD, n (%) | 21 (2.2) | 66 (17.4) | < 0.001 |
| CAD, n (%) | 17 (1.8) | 41 (10.8) | < 0.001 |
| HF, n (%) | 11 (1.2) | 31 (8.2) | < 0.001 |
| Malignancies, n (%) | 107 (11.4) | 59 (15.6) | 0.039 |
| Thyroid disease | 28 (3.0) | 15 (4.0) | 0.369 |
| Sepsis, n (%) | 142 (15.1) | 187 (49.3) | < 0.001 |
| Previous antidiabetic drugs, n (%) |  |  |  |
| α-glucosidase inhibitors, n (%) | 9 (1.0) | 12 (3.2) | 0.004 |
| Insulin secretagogues, n (%) | 11 (1.2) | 17 (4.5) | < 0.001 |
| Thiazolidinediones, n (%) | 1 (0.1) | 2 (0.5) | 0.147 |
| Metformin, n (%) | 10 (1.1) | 18 (4.7) | < 0.001 |
| Insulin, n (%) | 21 (2.2) | 22 (5.8) | 0.001 |
| Previous use of corticosteroids, n (%) | 86 (9.2) | 48 (12.7) | 0.057 |
| Admission type, n (%) |  |  | < 0.001 |
| Elective surgical, n (%) | 739 (78.8) | 138 (36.4) |  |
| Emergency surgical, n (%) | 67 (7.1) | 77 (20.3) |  |
| Medical, n (%) | 132 (14.1) | 164 (43.3) |  |
| Baseline serum creatinine, mg/dL | 0.70 (0.59-0.84) | 0.70 (0.57-0.97) | 0.182 |
| Baseline eGFR, mL/min/1.73 m^2^ | 103.4 (88.3-124.2) | 104.2 (72.2-136.0) | 0.493 |
| Serum creatinine at ICU admission, mg/dL | 0.78 (0.66-0.94) | 1.10 (0.84-1.46) | < 0.001 |
| Serum Cystatin C at ICU admission, mg/L | 0.78 (0.62-0.95) | 1.13 (0.83-1.57) | < 0.001 |
| HbA1c at ICU admission (%) | 5.6 (5.3-6.0) | 5.9 (5.5-6.4) | < 0.001 |
| Serum glucose at ICU admission (mg/dL) | 120.6 (103.6-146.0) | 144.0 (119.3-183.6) | < 0.001 |
| APACHE II score | 10 (7-13) | 17 (11-25) | < 0.001 |
| UP, ml/kg/h | 2.0 (1.6-2.6) | 1.9 (1.3-2.6) | 0.006 |
| Outcomes |  |  |  |
| Length of ICU stay, days | 2 (2-4) | 5 (2-10) | < 0.001 |
| Length of hospital stay, days | 11 (8-15) | 14 (9-23) | < 0.001 |
| RRT (during ICU stay), n (%) | 3 (0.3) | 18 (4.7) | < 0.001 |
| ICU mortality, n (%) | 16 (1.7) | 52 (13.7) | < 0.001 |
| In-hospital mortality, n (%) | 24 (2.6) | 60 (15.8) | < 0.001 |

**Abbreviation: AKI, Acute kidney injury; BMI Body mass index; DM, diabetes mellitus, including patient with known DM and patients without known DM but admission HbA1c ≥ 6.5% ; CAD, coronary artery disease; HF, heart failure; CKD, chronic kidney disease, defined as baseline eGFR< 60 ml/min per 1.73m^2^; eGFR, estimated glomerular ﬁltration rate; ACR, urinary albumin/creatinine ratio; Cre, creatinine concentration; HbA1c, Glycated hemoglobin; APACHE II, Acute Physiology and Chronic Health Evaluation score; UP, Urine production first 24 h after admission; ICU, Intensive care unit; RRT, renal replacement therapy.**

**The non-normally distributed continuous variables are expressed as median (25th percentile to 75th percentile [interquartile range]). Categorical variables are expressed as n (%).**
